# Beyond type 2 inflammation: An innate–myeloid axis is linked to recurrence of chronic rhinosinusitis with nasal polyps

**DOI:** 10.1016/j.jaci.2026.07.018

**Published:** 2026-08-07

**Authors:** Tiffany Dharia, Mabel Zawacki, Rie Maurer, Regan W. Bergmark, Stella E. Lee, Alice Z. Maxfield, Rachel E. Roditi, Pui Y. Lee, Evan Hsu, Courtney LeSon, Radomir Kratchmarov, Barbara Balestrieri, Tanya M. Laidlaw, Kathleen M. Buchheit

**Affiliations:** a the Division of Allergy and Clinical Immunology, Mass General Brigham, Boston; b Harvard Medical School, Boston; c the Department of Otolaryngology-Head and Neck Surgery, Mass General Brigham, Boston; d the Division of Immunology, Children’s Hospital, Boston.

**Keywords:** Aspirin-exacerbated respiratory disease, AERD, NSAID-ERD, chronic rhinosinusitis with nasal polyps, biomarker, interleukin-1 beta, interleukin-5 receptor alpha, CRSwNP, biologics, dupilumab

## Abstract

**Background:**

Chronic rhinosinusitis with nasal polyps (CRSwNP) is a heterogeneous inflammatory disease with variable outcomes after functional endoscopic sinus surgery (FESS). Predictive biomarkers for post-FESS CRSwNP recurrence remain poorly defined.

**Objective:**

We sought to identify tissue-based predictors of CRSwNP recurrence after FESS and assess dupilumab-induced modifications.

**Methods:**

From a cohort of patients with CRSwNP (including nonsteroidal anti-inflammatory drug–exacerbated respiratory disease) who underwent FESS, we retrospectively identified 91 who could be dichotomized by clinical outcome as post-FESS rapid recurrence (<1 year) or slow/no recurrence (>2 years without recurrence). Nasal polyp tissue was analyzed for 69 inflammatory mediators via proteomics and ELISA. Logistic regression with least absolute shrinkage and selection operator (aka LASSO) feature selection identified candidate predictors. In an independent, second prospective cohort of 17 CRSwNP patients initiating dupilumab, nasal fluid was collected at baseline and after 2 months of dupilumab therapy and profiled for the same mediators.

**Results:**

Twenty-three tissue mediators strongly predicted rapid CRSwNP recurrence (area under the curve > 0.70), including markers of type 2 (T2) inflammation (IL-4, IL-13, IL-5Rα, eosinophil cationic protein, IgE) and innate inflammation and/or myeloid involvement (CCL3/4/7/8/13, CSF2, IL-1β, IL-6, oncostatin M, MMP12, HIF-1α, TNF-α, TNFSF14). Of these, dupilumab significantly reduced IL-5Rα and several chemokines (CCL3, CCL4, CCL13) but had no effect on key myeloid mediators (CCL8, CSF2, IL-1β, MMP12, HIF-1α).

**Conclusions:**

Both T2 and non-T2 mediators play a role in rapid post-FESS recurrence in CRSwNP. T2 blockade with dupilumab reduces T2-associated mediators but does not suppress a parallel innate/myeloid inflammatory axis.

## Capsule summary

Nasal polyp tissue analysis from patients with chronic rhinosinusitis with nasal polyps (CRSwNP) identified multiple inflammatory mediators that spanned type 2 (T2), innate, and myeloid pathways and predicted rapid CRSwNP recurrence after functional endoscopic sinus surgery, indicating that persistent innate–myeloid signaling may contribute to CRSwNP recurrence, suggesting future targets for improved long-term disease management.

## GRAPHICAL ABSTRACT

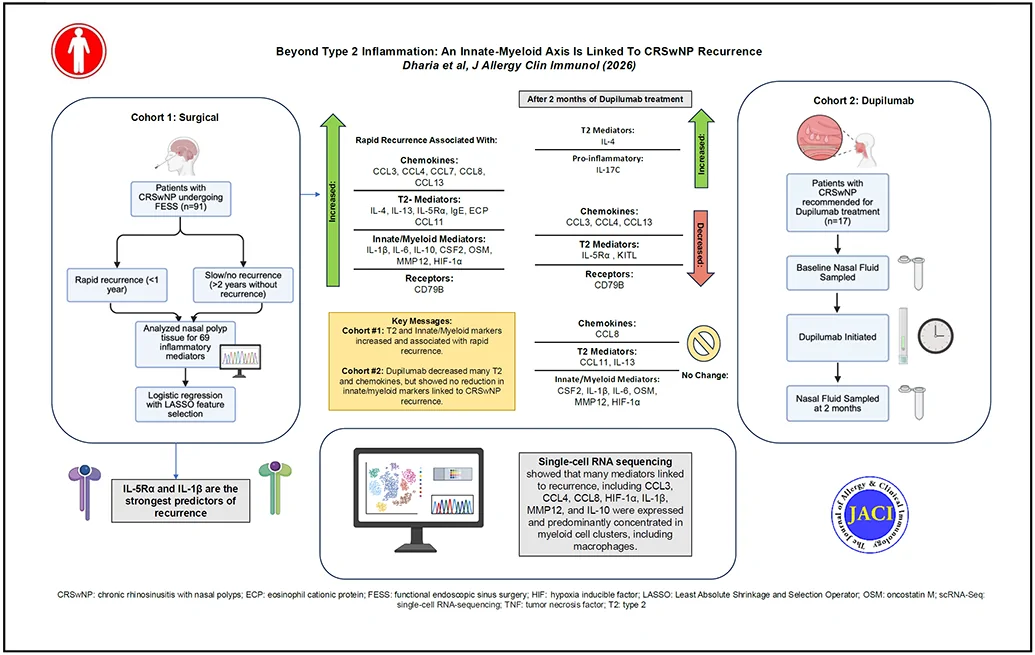

Chronic rhinosinusitis with nasal polyps (CRSwNP) is a chronic inflammatory disease of the upper airways characterized by nasal obstruction, loss of smell, and impaired quality of life.^1-4^ Functional endoscopic sinus surgery (FESS) is often pursued when medical management fails,^5,6^ but post-FESS recurrence of nasal polyps is not uncommon. It has been reported that symptom return and objective evidence of CRSwNP recurrence occurs within 12 months of surgery in 38% of patients,^7^ and long-term revision FESS rates for CRSwNP have been reported as 9.9% to 25%,^8-12^ raising the question of whether alternative medical management for that subset of patients would be preferable. With multiple US Food and Drug Administration– and European Medicines Agency–approved biologics for CRSwNP (omalizumab, mepolizumab, dupilumab, tezepelumab, and depemokimab)^13-17^ and additional biologic agents showing promise,^18^ a key clinical challenge is to determine which patients have disease that is likely to benefit from FESS versus instead benefiting from early initiation of biologic therapy.

The use of phenoendotypic factors in predicting CRSwNP treatment outcomes is increasingly recognized as critical to offering personalized management approaches.^19-26^ For example, patients with nonsteroidal anti-inflammatory drug (NSAID)-exacerbated respiratory disease (NSAID-ERD), also known as aspirin-exacerbated respiratory disease, the triad of asthma, CRSwNP, and respiratory reactions to cyclooxygenase-1 inhibitors represents a particularly high-risk group, with very high rates of CRSwNP recurrence after FESS and typically a more severe disease course even with substantial adjuvant medical management.^21,25,27-30^ Further, patients with CRSwNP and comorbid asthma generally have more severe CRSwNP and a greater likelihood of requiring revision FESS.^21,22,29-31^ The presence of both NSAID-ERD and asthma, linked to eicosanoid dysregulation, is strongly associated with increased need for biologic therapy among patients with CRSwNP.^32,33^ For this subgroup, predicting post-FESS recurrence is especially critical because surgery may only provide temporary benefit and the patients often have multiple indications for biologic therapy.

An extensive body of literature supports type 2 (T2) inflammation as a central driver of CRSwNP and NSAID-ERD, characterized by elevated tissue levels of IL-4, IL-5, IL-13, IgE, and eosinophilic infiltration, all of which correlate with more severe disease.^18,22,28,34-39^ However, recent data indicate that patients with a pure T2 endotype do not necessarily experience worse outcomes compared to those with a low inflammatory profile.^20^ Further, T2-targeted biologics are not uniformly effective, and none has proven to be curative.^13,40,41^ For example, although eosinophil infiltration into the sinus tissues is a hallmark of severe, T2-associated CRSwNP, we previously reported that patients treated with mepolizumab still experienced CRSwNP recurrence after FESS,^42^ underscoring that IL-5 and eosinophil blockade alone are insufficient to prevent recurrence.

Dupilumab, an IL-4Rα antagonist that blocks IL-4 and IL-13 signaling, has shown marked efficacy in rapidly reducing polyp burden and improving sinonasal symptoms, as reported in phase 3 trials and subsequent real-world studies, particularly in patients with NSAID-ERD.^13,43-46^ Yet when dupilumab is discontinued, CRSwNP symptoms and inflammation invariably recur, suggesting that although dupilumab temporarily controls symptoms, it is not inherently disease modifying.^13^

These clinical observations highlight a mechanistic conundrum: T2 inflammation is clearly central to symptoms but does not fully explain CRSwNP recurrence biology. Innate pathways—including epithelial-derived mediators, neutrophilic inflammation, and monocyte/macrophage-driven signals—are increasingly recognized in driving the underlying pathobiology of CRSwNP, even for patients with overlapping T2 disease or mixed endotypes.^19,20,24,47-50^ We therefore hypothesized that post-FESS CRSwNP recurrence is driven not solely by T2 pathways but also by innate inflammatory pathways, and that these mediators would remain active despite dupilumab treatment.

## METHODS

### Patient characterization

#### FESS cohort.

Patients with a history of CRSwNP whose disease remained inadequately controlled despite appropriate medical management and who were scheduled for FESS were recruited from the Brigham and Women’s Hospital (Boston, Mass) allergy/immunology and otolaryngology clinics between January 2015 and March 2022. The Mass General Brigham institutional review board approved the study, and all participants provided written informed consent.

**FIG 1.**
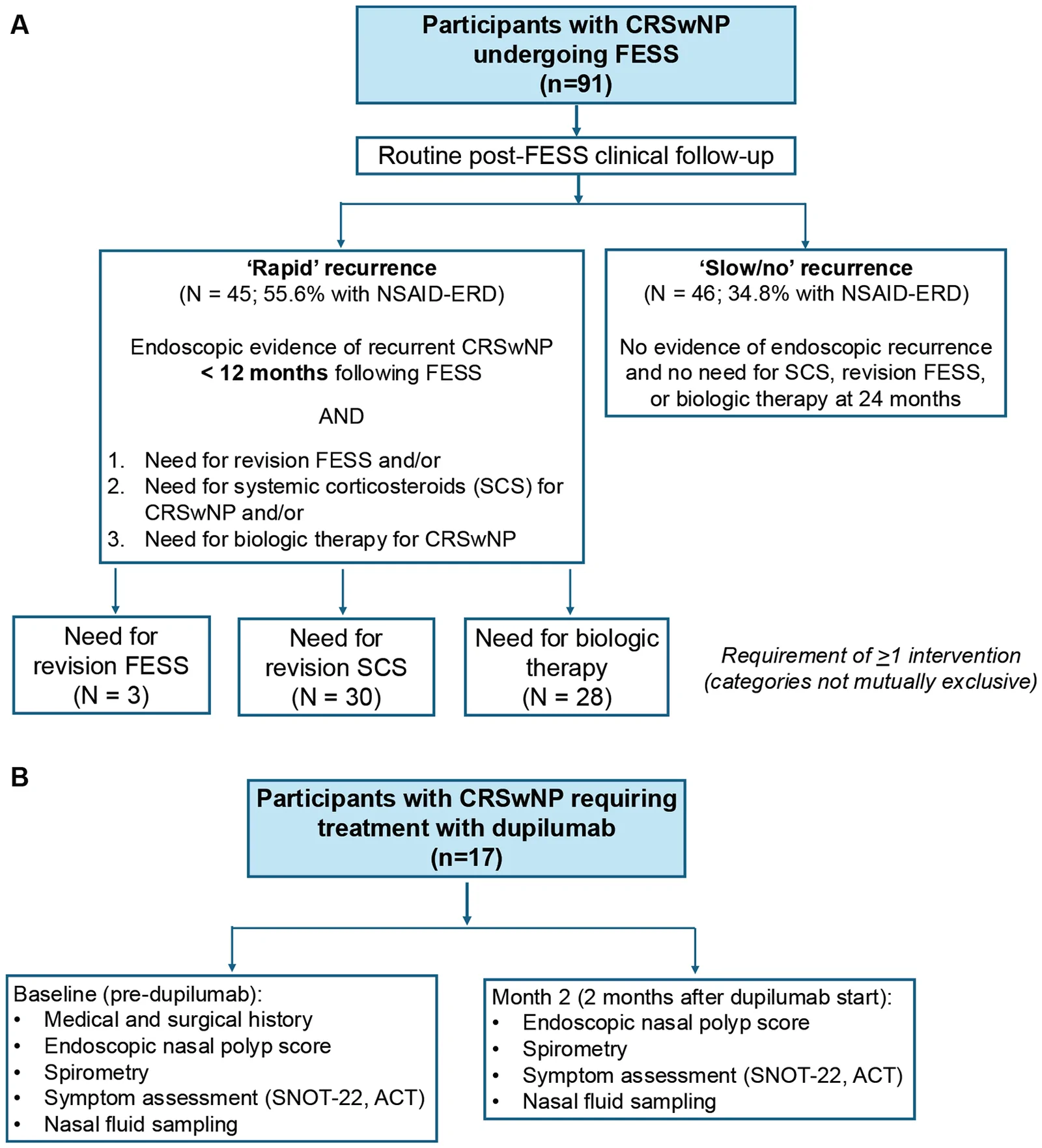
Study cohorts. **(A)** Flow diagram of surgical cohort. Ninety-one patients with CRSwNP who underwent FESS with tissue banking and clinical follow-up were identified. Patients were stratified into *rapid recurrence* (<1 year; n = 45) or *slow/no recurrence* (>2 years; n = 46) groups, with NSAID-ERD status recorded. **(B)** Flow diagram of dupilumab cohort. Seventeen patients with CRSwNP initiating dupilumab therapy were prospectively enrolled. Nasal fluid samples were collected at baseline (baseline) and after 2 months’ dupilumab treatment (month 2) for proteomic analysis.

Nasal polyp tissue was collected at the time of elective FESS from patients with physician-diagnosed NSAID-ERD (based on a history of asthma, CRSwNP, and respiratory reactions to cyclooxygenase-1 inhibitors) and patients with NSAID-tolerant CRSwNP (NT-CRSwNP). Those with unilateral polyps, allergic fungal rhinosinusitis, use of systemic corticosteroids or aspirin therapy after desensitization in the 1 month preceding FESS, or use of biologic therapy in the 6 months preceding FESS were excluded. Participants were followed for a minimum of 2 years after FESS to evaluate them for recurrence of CRSwNP. In this retrospective analysis, patients were defined as having rapid CRSwNP recurrence if they had endoscopic evidence of CRSwNP recurrence within 12 months after FESS and also met one of the following 3 criteria indicating a clinical need for escalation of treatment for inadequately controlled CRSwNP: (1) need for revision FESS for CRSwNP, (2) need for systemic corticosteroid treatment for CRSwNP, and/or (3) need for biologic treatment for CRSwNP. For comparison, we then selected patients with *slow/no CRSwNP recurrence,* defined as having no evidence of clinically significant recurrence at 24 months after FESS (Fig 1, A). Because of the retrospective nature of the study, standardized symptom scores, radiologic scores, and endoscopic scores (Sinonasal Outcome Test 22 [SNOT-22], Lund-Mackay, nasal polyp score) were not available for all patients and are not included in the definition of *recurrence.* All patients were prescribed intranasal corticosteroids after FESS, although we do not have any adherence data for intranasal corticosteroid receipt in this cohort. Data from the electronic medical record were collected regarding aspirin therapy after desensitization, biologic therapy, need for revision FESS, and requirement for corticosteroids at follow-up visits. Retrospective data including age, sex, race, history of atopy (physician-diagnosed allergic rhinitis, atopic dermatitis), cardiovascular disease, pre-FESS blood eosinophil count, and pre-FESS serum IgE were extracted from the electronic medical record.

#### Dupilumab cohort.

In an independent cohort, participants between 18 and 70 years old with CRSwNP were recruited from the Brigham and Women’s Hospital allergy/immunology clinics from 2023 to 2025. The Mass General Brigham institutional review board approved the study, and all participants provided written informed consent. Patients with CRSwNP who met the criteria for Food and Drug Administration–approved receipt of every-other-week dupilumab (300 mg provided subcutaneously) for the treatment of CRSwNP were prescribed dupilumab by their allergist/immunologist as part of their usual clinical care. Patients were recruited before initiation of their first dose of dupilumab. Participants were followed for this observational study for 2 months, and data are reported from a baseline visit before their first dose of dupilumab (baseline) and again after 2 months of dupilumab therapy (month 2) (Fig 1, B). Participants were excluded if they received any other biologic therapy within 6 months of enrollment.

### Nasal polyp tissue collection and nasal fluid collection

Nasal polyp tissue was collected at the time of elective FESS from patients with physician-diagnosed CRSwNP. For both CRSwNP and NSAID-ERD, polyp tissue originating from the ethmoid sinus was surgically excised and collected. Tissue was placed in RPMI 1640 (Corning, Corning, NY) with 10% fetal bovine serum (Thermo Fisher Scientific, Waltham, Mass) and 1 U/mL penicillin–streptomycin for transport to the laboratory on ice. Within 2 hours of surgery, the tissue was removed from RPMI 1640 and divided into segments. One segment was transferred into Cell Lytic M Cell Lysis Reagent (Sigma-Aldrich, St Louis, Mo) with 2% protease inhibitor (Roche, Indianapolis, Ind) for protein extraction, and the tissue was homogenized with a gentleMACS Dissociator (Miltenyi Biotec, San Diego, Calif). Polyp tissue lysate supernatants were stored at −80°C.

For patients undergoing treatment with dupilumab, nasal secretions were sampled separately from both nostrils with Nasosorption FX-R (Hunt Developments, Midhurst, United Kingdom), a noninvasive upper airway sampling method that uses a synthetic absorptive matrix to collect nasal mucosal lining fluid directly from the nasal mucosal surface. The strips were placed in 300 μL phosphate-buffered saline. The strips were then removed from their respective tubes and placed in a Costar Spin-X insert (Corning) and spun down. Nasal fluid supernatants were stored at −80°C.^51^

### Protein analyses

Polyp tissue lysates and nasal fluid from patients with NSAID-ERD and CRSwNP were analyzed for 66 protein analytes with the Olink Target 48 panel (Olink; Thermo Fisher Scientific) and a custom Olink 21 Flex panel, and via ELISAs for serum total IgE, eosinophil cationic protein (ECP), and neutrophil elastase (see Table E1 in this article’s Online Repository available at www.jacionline.org). Protein lysates were normalized to a concentration of 1 mg/mL for Olink analyses. Internal controls and calibrators were included with each run. Olink NPX Signature software was used for quality control and data analysis. Normalized protein concentrations were converted to absolute units (pg/mL) on the basis of an internal standard curve.^48^ Analytes below the limit of quantification for >50% of the samples were removed from the final analysis. Protein lysate supernatants from nasal polyp tissue homogenized as reported above were assessed by ELISA for Neutrophil Elastase (R&D Systems, Minneapolis, Minn), IgE (Abcam, Waltham, Mass), and ECP (Abcam). Total protein was measured with the Pierce BCA Protein Assay kit (Thermo Fisher Scientific), and all protein analyte levels were normalized to total protein in the polyp tissue lysate or nasal fluid.

### Single-cell RNA sequencing

Raw FASTQ files from a prior publicly available single-cell RNA sequencing (scRNA-Seq) study of healthy individuals (n = 5) and patients with heterogeneous chronic rhinosinusitis, previously classified as eosinophilic CRSwNP (n = 6), noneosinophilic CRSwNP (n = 5), and chronic rhinosinusitis without nasal polyps (n = 5),^52^ were obtained, and count matrixes were generated by Cell Ranger (10x Genomics, Pleasanton, Calif). We removed low-quality cells with <200 unique transcripts and a high percentage of mitochondrial transcripts (>15%). After these quality control metrics, a total of 115,856 cells were analyzed. All subsequent steps were performed using standard functions in Seurat software (NormalizeData, FindVariableFeatures, ScaleData, RunPCA, FindNeighbors, FindClusters, RunU-MAP) with default parameters and a resolution of 0.2 for coarse cell typing. The algorithm Harmony was used for integration^53^ of each donor to remove batch effect, with parameter λ = 1. The analysis package scCustomize^54^ was used for generation of FeaturePlots.

### Statistical analysis

Data analysis was completed by SAS v9.4 software (SAS Institute, Cary, NC) and GraphPad Prism (GraphPad Software, La Jolla, Calif). Differences between patients with rapid and slow/no recurrence were analyzed by *t* test or Mann-Whitney *U* test for continuous variables or by chi-square test for categorical variables, as appropriate. All candidate biomarkers were log transformed. We first identified candidate biomarkers detectable with *c* statistic > 70% using univariate logistic regression. We then performed least absolute shrinkage and selection operator (LASSO) regression to identify predictive tissue biomarkers for rapid CRSwNP recurrence. Our sample was split into half into training and validation sets. The HPGENSELECT procedure with method = LASSO was applied. The HPGENSELECT procedure constructs a data-driven sequence of regularization parameters (λ), beginning at the maximum value for which all coefficients are zero and decreasing along the solution path. At each step, the model was refit and evaluated by using a validation partition. Model selection was based on the “choose = validate” criterion, which selects the λ that minimizes the validation average squared error (see Fig E1 in the Online Repository available at www.jacionline.org). The variables selected by LASSO regression were further investigated to create the final predictive model by performing multivariable logistic regression and the receiver-operating characteristic curves. For the paired on- and off-dupilumab samples, a paired *t* test or Wilcoxon matched-pairs signed-rank test was used, as appropriate.

## RESULTS

### Demographic and clinical characteristics

We first identified a surgical cohort of 91 patients with CRSwNP who underwent FESS with tissue banking and had sufficient follow-up to classify their recurrence course. Patients’ disease was retrospectively dichotomized as rapid recurrence (<1 year; n = 45) or slow/no recurrence (>2 years; n = 46), with a cohort of 91 patient samples chosen from our larger set of banked samples so that we would have roughly equal numbers of patients in the rapid versus slow/no recurrence subgroups. For participants to be included in the rapid recurrence group, we identified patients with endoscopic evidence of CRSwNP recurrence and a substantial increase in CRSwNP symptoms requiring one or more significant interventions. Because several patients required multiple overlapping treatments, the interventions for these 45 unique patients included revision FESS (n = 3), systemic corticosteroids for treatment of CRSwNP (n = 30), and/or need for biologic therapy for CRSwNP (n = 28). More than half of the participants in the rapid recurrence group had a diagnosis of NSAID-ERD, and more than 75% of participants in the rapid recurrence group had comorbid asthma. Participants with rapid recurrence also had significantly higher baseline blood eosinophil counts and serum IgE levels than those with slow recurrence (Fig 1, A and Table I).

The second prospective cohort of 17 adults with CRSwNP, of which 70% had a diagnosis of NSAID-ERD and 88% had prior FESS, was enrolled at the initiation of dupilumab therapy, with nasal fluid collected at baseline and after 2 months of dupilumab treatment (Fig 1, B). Participants had poorly controlled sinonasal symptoms at baseline with improvement after 2 months of dupilumab treatment, as evidenced by improved SNOT-22 score, University of Pennsylvania Smell Identification Test (aka UPSIT), nasal polyp scores, and peak nasal inspiratory flow (see Table E2 in the Online Repository available at www.jacionline.org). Demographic and clinical characteristics of both cohorts are presented in Tables I and II.

### Nasal polyp tissue predictors stratified by disease and recurrence status

**FIG 2.**
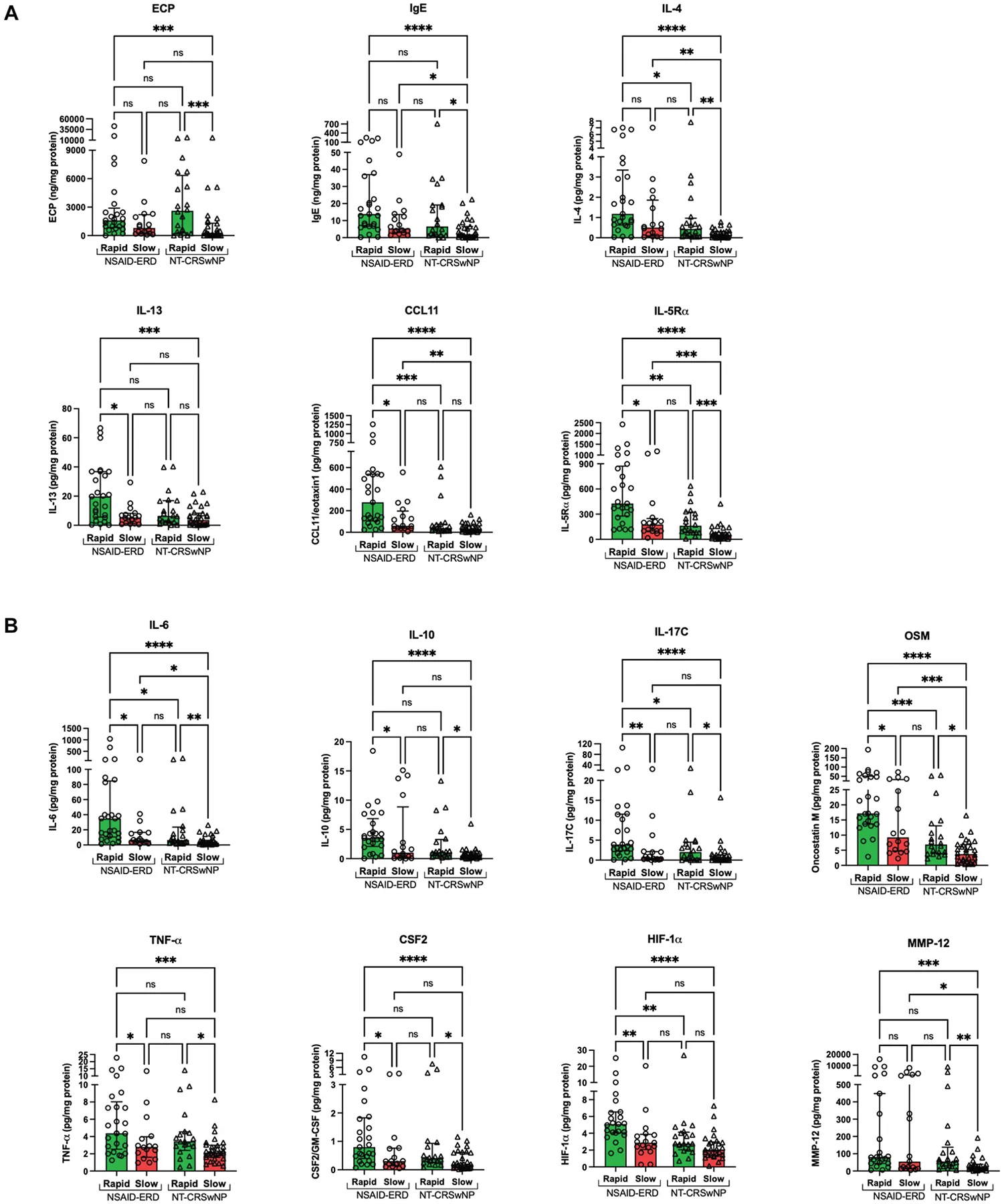
Selected nasal polyp levels of predictive mediators stratified by disease status and CRSwNP recurrence. Nasal polyp tissue concentrations of selected recurrence-associated mediators in patients with NSAID-ERD or NT-CRSwNP who experienced rapid or slow/no post–endoscopic sinus surgery recurrence are shown. Selected mediators associated with T2 inflammation **(A)** and mediators associated with monocyte and macrophage-mediated and innate inflammation **(B)**. Data are shown as medians and interquartile ranges. *P* values calculated by Wilcoxon signed-rank or *t* test, as appropriate: *P < .05, **P < .01, ***P < .001, ****P < .0001.

Proteomic analysis of surgically excised nasal polyp tissue revealed 23 log-transformed mediators with strong discriminatory power for predicting CRSwNP recurrence (*c* > 0.70) (Table III). We compared tissue levels of these 23 mediators across patient subsets defined by both recurrence rate and NSAID-ERD status. Many mediators were elevated in both NSAID-ERD and NT-CRSwNP patients with rapid recurrence compared to their slow/no recurrence counterparts (Fig 2, and see Fig E2 in the Online Repository available at www.jacionline.org). Notably, high tissue levels of chemokine C-C motif ligand (CCL) 8, IL-1β, IL-6, granulocyte-macrophage colony-stimulating factor (CSF2; aka GM-CSF), IL-10, and oncostatin M (OSM) were consistently associated with rapid regrowth across both patient groups, suggesting that innate/myeloid markers of recurrence are shared across CRSwNP subtypes and are not limited to NSAID-ERD (Fig 2, B, and Fig E2, A).

In addition to the differences noted across groups in mediators associated with myeloid/macrophage and innate inflammation, there were significant differences in mediators associated with T2 inflammation including IL-4, IL-13, IL-5Rα, and IgE in patients with rapid versus slow/no recurrence in one or both of the disease states (NSAID-ERD vs NT-CRSwNP) (Fig 2, A). There were also significant differences in the B-cell antigen receptor complex CD79B, TNF receptor superfamily, member 4 (aka TNFRSF4)/OX40L, and TNF superfamily member 14 (TNFSF14)/LIGHT (Fig E2, B).

### Dupilumab-induced changes in nasal fluid mediators

**FIG 3.**
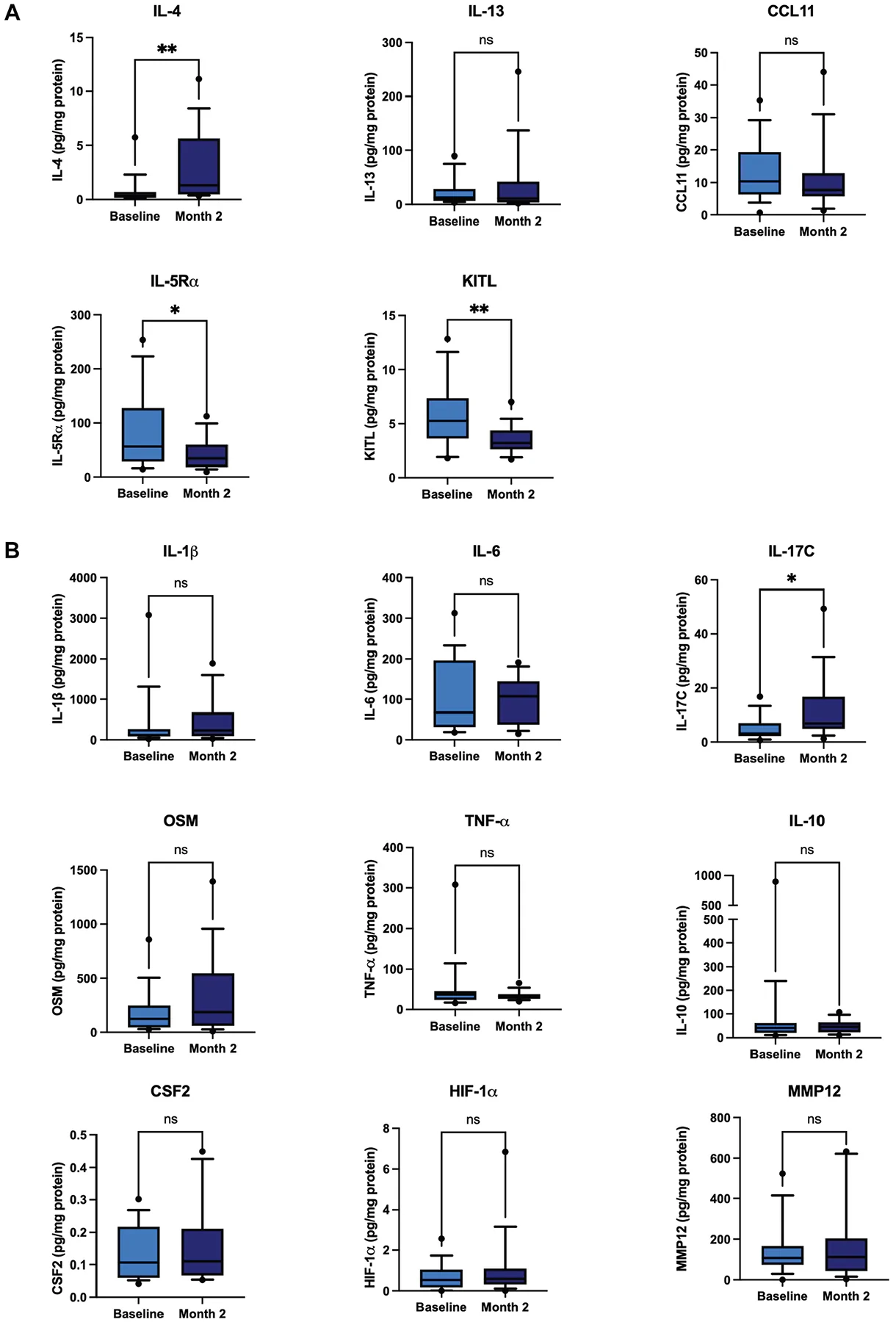
Dupilumab-induced changes in recurrence-associated mediators in nasal fluid. Paired analyses of nasal fluid concentrations at baseline and after 2 months of dupilumab therapy (month 2) in 17 CRSwNP patients. Shown are selected mediators identified as predictors of postsurgical recurrence, including mediators associated with T2 inflammation **(A)** and innate/myeloid mediators **(B)**. *Horizontal lines* indicate medians; *boxes,* interquartile range; and *whiskers,* 10th to 90th percentiles. *P* values from paired *t* test or Wilcoxon signed-rank test: *P < .05, **P < .01, ***P < .001.

In the dupilumab cohort, paired nasal fluid analyses demonstrated selective suppression of T2-associated mediators after 2 months of dupilumab treatment. IL-5Rα nasal fluid levels decreased by nearly half, and several T2-associated chemokines (CCL3, CCL4, CCL13) also declined significantly (Fig 3, A, and see Fig E3, A, in the Online Repository available at www.jacionline.org). In contrast, key recurrence-associated innate/myeloid mediators identified in the post-FESS recurrence cohort, including CCL8, CSF2, IL-1β, IL-6, OSM, matrix metalloproteinase (MMP) 12, and HIF-1α, were not reduced by dupilumab. Moreover, IL-17C (Fig 3, B) and IL-4 (Fig 3, A) levels increased during therapy (Fig 3, A and B). There was also a significant decrease in CD79B after 8 weeks’ dupilumab treatment (Fig E3, B).

### Cellular localization of markers associated with recurrence identified by scRNA-Seq

**FIG 4.**
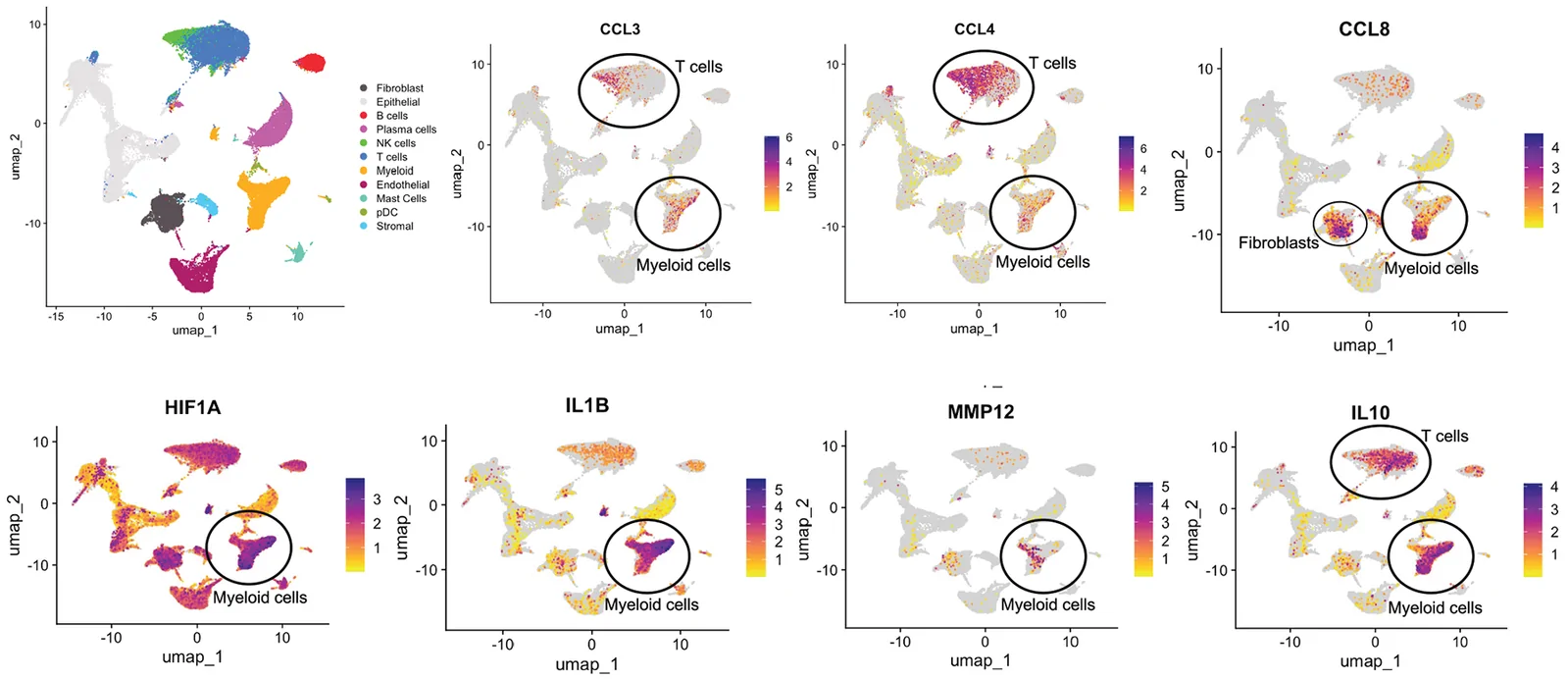
Sinus tissue scRNA-Seq. Uniform manifold approximation and projection (UMAP) plots of surgically excised sinus tissue cells displaying cell clusters identified within sinus tissues *(top left)* and their expression of CCL3, CCL4, CCL8, HIF1A, IL1B, MMP12, and IL10 transcript.

To pinpoint the cellular sources of mediators most associated with rapid recurrence within the nasal polyp environment, we reanalyzed a scRNA-Seq dataset of cells from surgically resected CRSwNP tissue.^52^ Among the transcripts linked to rapid CRSwNP recurrence, including CCL3, CCL4, CCL8, HIF1A, IL1B, MMP12, and IL10, expression was predominantly concentrated in myeloid cell clusters, which includes macrophages (Fig 4, and see Fig E4, A, in the Online Repository available at www.jacionline.org). Notably, IL1B, which had emerged as a strong signal in the multivariable logistic regression model (see Fig E5, B, in the Online Repository), was also most highly expressed within these myeloid cell populations (Fig E4, B). CCL3, CCL4, and IL10 were also expressed in nasal polyp T cells, whereas CCL8 was also expressed in nasal polyp fibroblasts (Fig E4, A).

We have previously shown that IL-5Rα is expressed on multiple cells in nasal polyp tissue, including antibody-secreting cells and epithelial cells.^28,55^ We correlated lysate ECP, CD79B, and epidermal growth factor protein levels with IL-5Rα levels and found that IL-5Rα positively correlated with ECP and CD79b but not with epidermal growth factor (see Fig E6 in the Online Repository available at www.jacionline.org).

### Predictors of CRSwNP recurrence

Of the 23 strongly predictive mediators, 6 are generally considered to be T2 associated (IL-4, IL-13, IL-5Rα, IgE, ECP, CCL11/eotaxin-1), supporting their central role in CRSwNP biology. Levels of the alarmins, IL-33 and thymic stromal lymphopoietin, and IL-5 were not predictive of recurrence (see Fig E7 in the Online Repository available at www.jacionline.org). In contrast, the majority of the predictive mediators were innate and myeloid-related signals, including chemokines (CCL3, CCL4, CCL7, CCL8, CCL13), cytokines (IL-1β, IL-6, IL-10, IL-17C, TNF-α, OSM), growth/survival factors (CSF2, hypoxia-inducible factor [HIF]-1α), and tissue remodeling enzymes (MMP12) (Table III).

To address multiplicity and develop a predictive model for CRSwNP recurrence, we applied LASSO and logistic regression to identify the markers most strongly associated with post-FESS CRSwNP recurrence. LASSO regression identified 9 tissue biomarkers linked to rapid CRSwNP recurrence: log CCL8, log CCL11, log IL-6, log IL-17C, log IL-1β, log IL-5Rα, log ECP, log IL-10, and log IL-13. A final model after multivariable logistic regression identified that together, log IL-5Rα and log IL-1β formed the strongest predictive model of recurrence with an area under the curve (95% confidence interval) of 0.85 (0.77-0.93) (Table IVand Fig E5, A). There was no change when adjusting for the disease status (NSAID-ERD vs NT-CRSwNP), indicating that both biomarkers are representative predictors of post-FESS recurrence regardless of disease status (Table IV and Fig E5, B). Notably, there were significantly higher levels of IL-5Rα and IL-1β in the NSAID-ERD total cohort (rapid and slow/no recurrence) compared to the NT-CRSwNP total cohort (rapid and slow/no recurrence) (*P* < .0001 and .0076, respectively; data not shown).

## DISCUSSION

In this study, we identified a panel of inflammatory mediators in nasal polyp tissue that strongly predict poor post-FESS outcomes in patients with CRSwNP. Consistent with prior reports, several markers of T2 inflammation, such as IL-4, IL-13, IL-5Rα, ECP, and IgE, were associated with CRSwNP recurrence.^22,28,56-58^ However, we also observed strong signals from mediators linked to innate and myeloid activation and recruitment, including multiple chemokines (CCL3, CCL4, CCL7, CCL8, CCL13), proinflammatory cytokines (IL-1β, IL-6, TNF-α, OSM, CSF2), and tissue homeostasis (IL-10, MMP12, HIF-1α). This integrated inflammatory signature suggests that while T2 inflammation remains central to CRSwNP pathogenesis, recurrent disease is driven by a broader network of immune activation that extends beyond canonical T2 and eosinophilic inflammation. The individual mediators that distinguish between rapid CRSwNP recurrence and slow/no recurrence further demonstrated that the innate/myeloid axis is not simply an NSAID-ERD–specific phenomenon but rather a broader driver of disease severity. While NSAID-ERD patients were overrepresented among rapid CRSwNP recurrences, the same mediators also predict poor outcomes in NT-CRSwNP. Though NSAID-ERD is often mechanistically distinguished by eicosanoid imbalance, the association of innate/myeloid and T2 mediators with recurrence suggests that these markers reflect broader mechanisms of disease persistence and severity rather than NSAID-ERD–specific biology alone. Both T2 and innate/myeloid markers predicted rapid CRSwNP recurrence, with IL-5Rα and IL-1β together as the most predictive model of recurrence in our cohort, regardless of aspirin sensitivity.

Consistent with current paradigms, our data confirm that several T2-associated mediators are key predictors of postoperative recurrence. These cytokines and effector molecules are thought to drive the tissue eosinophilia, mucosal edema, and barrier dysfunction that underlie patients’ symptoms in CRSwNP.^13-15,59^ IL-5Rα, which is known to be expressed on multiple immune cells and epithelial cells in nasal polyp tissue,^55^ may play a role in driving eosinophilic inflammation, but it also could drive epithelial–mesenchymal transition and antibody-secreting cell-mediated inflammation, and we see that it correlates with both eosinophil and B-cell markers.^28,60,61^ Clinically, IL-4Rα blockade with dupilumab rapidly improves nasal obstruction, olfaction, and overall symptom burden, reflecting its potent inhibition of this T2 axis. The dupilumab-induced reduction of nasal fluid levels of IL-5Rα and eosinophil-associated chemokines such as CCL13 we observed after 2 months of treatment mirrors the rapid dupilumab-induced improvement in sinonasal symptoms consistently reported in phase 3 trials and observational studies.^13,43,45,62^ Taken together, these results reaffirm that T2 inflammation is essential to driving CRSwNP symptom burden and that blocking IL-4 and IL-13 signaling is highly effective for disease control. However, our new data underscore that dupilumab’s effects, while often dramatic for nasal symptom reduction, do not reflect resolution of the underlying chronic inflammatory program that promotes tissue remodeling and polyp regrowth. The underlying mechanisms that work to drive CRSwNP recurrence once dupilumab is withdrawn require further investigation.

Several of the mediators that most powerfully predicted rapid CRSwNP recurrence were chemokines and cytokines that recruit myeloid cells, particularly monocytes and macrophages. CCL3, CCL4, CCL7, and CCL8 each exhibited high discriminative ability (*c* = 0.71-0.77), consistent with prior reports implicating macrophage-rich infiltrates in CRSwNP recurrence and corticosteroid resistance.^63,64^ These chemokines act on C-C chemokine receptor (CCR) 1, CCR2, and CCR5, which mediate monocyte extrusion from bone marrow and migration to specific tissues. Once in the tissue, monocytes are then polarized toward inflammatory macrophages capable of producing HIF-1α, IL-1β, TNF-α, IL-6, OSM, and MMP12, all of which were also associated with rapid CRSwNP recurrence in our dataset. Together, these findings suggest that CRSwNP tissue prone to severe inflammation and post-FESS recurrence harbors an entrenched stromal–myeloid inflammatory axis that may sustain chronic inflammation and tissue remodeling independent of T2 cytokine signaling.^65^ The identification of HIF-1α among the top predictors further supports the concept that the recurrent polyp microenvironment is hypoxic and metabolically dysregulated, favoring proangiogenic remodeling, macrophage persistence, and epithelial dysregulation, in a microenvironment no longer dominated by T2 cytokines. This aligns with recent evidence demonstrating that a defective HIF-1α–CD73–adenosine signaling axis impairs wound healing and barrier dysfunction.^66^ Furthermore, HIF-1α activation of nucleotide oligomerization domain-like receptor protein 3 (aka NLRP3) in nasal epithelial cells drives release of IL-1β, leading to neutrophil recruitment, in a feed-forward loop driving inflammation, obstruction, and hypoxia.^67^ Notably, the upregulated myeloid signals were shared across both NSAID-ERD and NT-CRSwNP, suggesting a common mechanism of CRSwNP recurrence that extends beyond T2 biology. This observation expands on prior studies by highlighting the overlooked importance of monocyte/macrophage-driven inflammation in CRSwNP chronicity.

Our analysis of nasal fluid from paired pre- and post-dupilumab samples reinforces the hypothesis that ongoing epithelial–myeloid inflammation contributes to disease chronicity and CRSwNP recurrence. While dupilumab effectively suppressed some T2-associated mediators, it had no measurable effect on many of the non-T2 mediators most predictive of rapid CRSwNP recurrence, including CCL7, CCL8, CSF2, IL-1β, OSM, TNF-α, MMP12, and HIF-1α.^68^ Furthermore, dupilumab increased IL-4, likely due to blockade of IL-4Rα subsequently leading to accumulation of unbound IL-4 in the circulation, consistent with prior studies showing elevated levels of IL-4 in patients who received dupilumab treatment.^48^ The persistent myeloid activation may reflect an immune recalibration toward innate or myeloid pathways once T2 signaling is suppressed. Clinically, the continued activity may explain why dupilumab discontinuation is often followed by CRSwNP symptom recurrence and polyp regrowth, while T2 cytokine-driven edema and mucous production subside during treatment. The persistent myeloid inflammatory loop remains active and capable of reinitiating the cycle of tissue remodeling once IL-4/IL-13 blockade is removed. We hypothesize that in severe CRSwNP, inflammatory macrophages, in addition to other upstream cells and mediators including mast cells and dysregulated epithelium, persist as key upstream orchestrators of disease, inducing alarmin release, recruiting eosinophils and T_H_2 cells, and facilitating group 2 innate lymphoid cell/T_H_2 expansion. In this framework, macrophages are not merely downstream responders to established T2 inflammation but rather amplifiers that establish, and can rapidly reestablish after dupilumab withdrawal, the inflammatory niche that supports both T2 and non-T2 immune activation. In this context, dupilumab can be viewed as a highly effective symptomatic therapy but not a curative intervention.

Our findings highlight the need to broaden our therapeutic offerings beyond T2 inflammation in CRSwNP and may have immediate clinical implications. Preoperative biomarker profiling of CRSwNP tissue may help identify patients with strong myeloid-inflammatory signatures who are at high risk for post-FESS recurrence, as well as those who may be good surgical candidates. Mechanistic targeting of macrophage activation pathways, such as CSF2, TNF-α, IL-1β, or hypoxia signaling, or combined inhibition of T2 and myeloid axes, could represent the next horizon for more comprehensive control of the biological processes that drive tissue remodeling and recurrence. Identifying individuals with elevated innate/myeloid signals could guide shared decision-making with patients when choosing between surgical and medical management, offering a personalized and potentially cost-effective management approach. Future studies dissecting the triggers for myeloid-associated mediators identified here, such as OSM, MMP12, and HIF-1α, could provide actionable targets beyond symptom suppression to complement existing biologics.

Our study is limited in that we utilized banked sinus tissue from participants undergoing FESS in a real-world setting. Patient-specific behavioral factors are known to influence post-FESS outcomes, specifically adherence to intranasal corticosteroid regimens, which could play a factor in recurrence in our cohort, as this was not a controlled trial with specific intranasal corticosteroid requirements and adherence monitoring.^69,70^ Further, because of the retrospective nature of the study, patients were followed according to usual clinical care, and a 2-year retrospective window was applied uniformly to assess for recurrence events without predefined follow-up, so it is possible that earlier recurrences could be missed in some patients. To mitigate this limitation, participants were dichotomized into those with very rapid versus slow/no recurrence after FESS. Therefore, this cohort was enriched for participants with NSAID-ERD (56% of participants with rapid recurrence) and with particularly severe disease and excludes participants who may have more modest rates of recurrence, limiting the generalizability of our findings to the general CRSwNP population. A key limitation of this study is its retrospective design, which precluded the use of standardized symptom, radiologic, and endoscopic scoring systems (SNOT-22, Lund-Mackay, nasal polyp score) to define recurrence, as these were not uniformly captured in the electronic medical record. Thus, we captured clinically meaningful recurrence by need for revision surgery or pharmacologic escalation. Future prospective studies using these scoring systems would be needed to validate our findings. Additionally, in the dupilumab cohort, the modest sample size and limited follow-up period constrain interpretation of longer-term immunologic trajectories, and extended observation will be necessary to determine the persistence of these effects. Therefore, biomarker panels that combine T2 and innate signals, such as IL-5Rα plus IL-1β, warrant validation in prospective cohorts of patients undergoing FESS. Such tools could guide individualized therapy, ensuring that high-risk patients could be offered biologics earlier in their disease course.

Another limitation of this study is that protein measurements were derived from two distinct compartments: nasal lysates for the post–endoscopic sinus surgery recurrence cohort and nasal fluid for the dupilumab cohort. While we do not have direct paired validation demonstrating that protein levels in lysates and fluid quantitatively correlate, existing literature suggests that many of these inflammatory mediators are elevated in both nasal tissue and nasal secretions, supporting their biological relevance across compartments.^71-73^ Nonetheless, the lack of direct cross-compartment correlation remains an important limitation of our study and area for future investigation and formal validation. Lastly, post-dupilumab discontinuation mediator data are not available in this cohort, though this represents a key area for future study.

Collectively, our data reinforce 3 major concepts: (1) T2 inflammation is central to CRSwNP symptom burden and is readily suppressed by dupilumab; (2) innate/myeloid pathways, including chemokine-driven monocyte recruitment, CSF2–mediated macrophage survival, and IL-1β-, OSM-, and MMP-driven remodeling, are critical markers of recurrence; and (3) targeting both arms of inflammation with medical or surgical management is imperative to CRSwNP disease remodeling. This integrated view explains why biologics that target only T2 pathways are highly effective but not curative. Candidate approaches include combining IL-4Rα blockade with interventions that reduce monocyte recruitment (eg, CCL8 axis), macrophage survival (CSF2/CSF1R inhibitors), or IL-1β/OSM signaling. By identifying the innate/myeloid axis as a key link to CRSwNP recurrence, our study provides both biomarkers for risk stratification and new potential targets for therapeutic innovation in CRSwNP.

## Supplementary Material

Supplemental Table E2

MMC5

MMC3

Supplemental Table E1

MMC4

figsE6

figsE7

figsE5

figsE1

figsE2

figsE4

figsE3

## Figures and Tables

**TABLE I.** Baseline participant characteristics for participants undergoing FESS

Characteristic	Rapid recurrence (n = 45)	Slow/no recurrence (n = 46)	*P* value
Age (years), mean ± SD	47.7 ± 15.9	49.4 ± 14.6	NS
Female sex	23 (51.1%)	21 (45.60%)	NS
NSAID-ERD	25 (55.6%)	16 (34.8%)	<.05
Blood eosinophil count (cells/μL)	570 (435-910)	280 (180-460)	<.0001
Serum IgE (IU/mL)	221.5 (76.5-395)	76 (32-186)	<.0001
Asthma	35 (77.8%)	22 (47.8%)	<.01
Cardiovascular disease	22 (47.8%)	16 (35.6%)	NS
Autoimmune disease	3 (6.5%)	3 (6.7%)	NS
Allergic rhinitis	17 (37.8%)	20 (44.4%)	NS

Unless otherwise noted, categorical variables are reported as frequencies and percentages, numerical variables, or median (Q1-Q3). *NS,* Not significant.

**TABLE II.** Baseline participant characteristics for participants undergoing dupilumab treatment

Characteristic	Dupilumab treatment (n = 17)
Age (years), mean ± SD	51.4 ± 17.4
Female sex	10 (58.8%)
NSAID-ERD	12 (70.6%)
Blood eosinophil count (cells/μL)	405 (265-473)
Asthma	14 (82.3%)
SNOT-22 score	41 (26.2-61)
ACQ-6 score	1.17 (0.17-2)
Allergic rhinitis	12 (70.5%)
Daily aspirin therapy after desensitization	4 (23.5%)
History of FESS	15 (88%)

Unless otherwise noted, categorical variables are reported as frequencies and percentages, numerical variables, or median (Q1-Q3). *ACQ-6,* Six-question Asthma Control Questionnaire.

**TABLE III.** Predictors of polyp recurrence in FESS cohort

Characteristic	Protein	*P* value from logistic regression	*c* statistic	Functional role
Myeloid cell chemotaxis	CCL3	.0004	0.756	Released by activated macrophages; recruits monocytes, T cells, eosinophils, and neutrophils
	CCL4	.0002	0.767	Released by activated macrophages and structural cells; recruits macrophages, T cells, and eosinophils
	CCL7	.0009	0.710	Released by activated macrophages and structural cells; recruits monocytes, macrophages, and neutrophils
	CCL8	.0002	0.745	Released by activated macrophages and structural cells; recruits monocytes, macrophages, and T cells
	CCL13	.0014	0.700	Released by activated macrophages and structural cells; recruits monocytes, macrophages, eosinophils, and T cells
T2 inflammation	ECP	.0029	0.731	Released by eosinophils; promotes inflammation
	IgE	.0035	0.713	Promotes mast cell degranulation and eosinophil activation
	IL-4	.0005	0.746	Promotes eosinophilic infiltration, IgE production, and tissue remodeling
	IL-13	.0027	0.702	Promotes tissue remodeling, mucus hypersecretion, and eosinophilic inflammation
	CCL11	.0007	0.700	Induces eosinophil chemotaxis
	IL-5Rα	<.0001	0.812	Receptor for IL-5 (drives eosinophilic inflammation)
Receptor/signaling	CD79B	.0005	0.733	B-cell antigen receptor complex
	TNFRSF4	.0005	0.743	OX40 receptor; plays role in T1 and T2 inflammation
	TNFSF14	.0005	0.729	Member of TNF receptor superfamily; involved in innate and adaptive processes
Innate/proinflammatory cytokines	IL-1β	.0002	0.777	Released by monocytes/macrophages; important in neutrophil recruitment and amplification of T1 inflammation
	IL-6	.0001	0.758	Released by multiple immune cells including monocytes/macrophages; important for leukocyte recruitment; enhances acute-phase response
	IL-17C	.0082	0.744	Released by epithelial cells; drives proinflammatory response
	OSM	.0001	0.759	Released by neutrophils, monocytes/macrophages, T cells, and IL-6 family cytokines; drives acute phase response; plays role in barrier dysfunction
	TNF-α	.0018	0.722	Released by monocytes/macrophages; proinflammatory cytokine
Regulatory cytokine	IL-10	.0012	0.737	Released by regulatory T cells and macrophages; has anti-inflammatory/tolerogenic cytokine
Growth/survival factors	CSF2	.0003	0.728	CSF2 attracts and stimulates survival of granulocytes, macrophages, and monocytes.
	HIF-1α	.0007	0.755	Transcription factor that regulates oxygen metabolism, angiogenesis, and cell survival
Tissue remodeling enzyme	MMP12	.0083	0.707	Macrophage-derived proenzyme with role in breaking down extracellular matrix

List of 23 nasal polyp tissue mediators identified by logistic regression with *c* statistics (AUC) > 0.70 for predicting rapid recurrence after FESS. Includes cytokines, chemokines, growth factors, and remodeling enzymes, with annotation of functional category (T2 vs innate, myeloid, and epithelial). All proteins were significantly elevated in patients with rapid recurrence compared to patients with slow/no recurrence. *AUC,* Area under the curve; *T1,* type 1; *TNFSF14,* TNF superfamily member 14.

**TABLE IV.** Multivariable logistic regression model

Predictor	OR (95% CI)	*P* value	AUC (95% CI)
Log(IL-5Rα)	7.58 (2.31-24.82)	.0008	0.85
Log(IL-1β)	4.60 (1.37-15.46)	.0135	(0.77-0.93)
Log(IL-5Rα)	12.66 (3.02-52.99)	.0005	0.85
Log(IL-1β)	5.29 (1.54-18.19)	.0083	(0.77-0.93)
Disease status, NSAID-ERD vs NSAID-tolerant CRSwNP	0.40 (0.12-1.37)	.1441	

*AUC,* Area under the curve; *CI,* confidence interval; *OR,* odds ratio.

## Key messages

- Rapid post–endoscopic sinus surgery recurrence of CRSwNP is not fully explained by T2 inflammation; innate and myeloid mediators strongly predict recurrence risk.
- Dupilumab effectively suppresses T2-driven markers but does not reduce innate/myeloid mediators associated with CRSwNP recurrence, providing a mechanistic explanation for disease recurrence after treatment cessation.
- Prospective biomarker profiling may identify high-risk patients with disease unlikely to benefit from FESS, and combined blockade of T2 and innate/myeloid pathways could represent the next horizon for durable CRSwNP treatment.

## Abbreviations used

CCL: Chemokine C-C motif ligand
CCR: C-C chemokine receptor
CRSwNP: Chronic rhinosinusitis with nasal polyps
CSF2: Granulocyte-macrophage colony-stimulating factor (aka GM-CSF)
ECP: Eosinophil cationic protein
FESS: Functional endoscopic sinus surgery
HIF: Hypoxia-inducible factor
LASSO: Least absolute shrinkage and selection operator
MMP: Matrix metalloproteinase
NSAID: Nonsteroidal anti-inflammatory drug
NSAID-ERD: NSAID-exacerbated respiratory disease
NT: NSAID tolerant
OSM: Oncostatin M
scRNA-Seq: Single-cell RNA sequencing
SNOT-22: Sinonasal Outcome Test 22
T2: Type 2
