# Supplementary material for: Beyond type 2 inflammation: An innate–myeloid axis is linked to recurrence of chronic rhinosinusitis with nasal polyps
Source: J Allergy Clin Immunol. Author manuscript; Available in PMC 2026 Sep 24. (PMC13606457; doi:10.1016/j.jaci.2026.07.018)
Supplement: Supplementary file 1 [file NIHMS2203575-supplement-MMC3.pdf]

A.

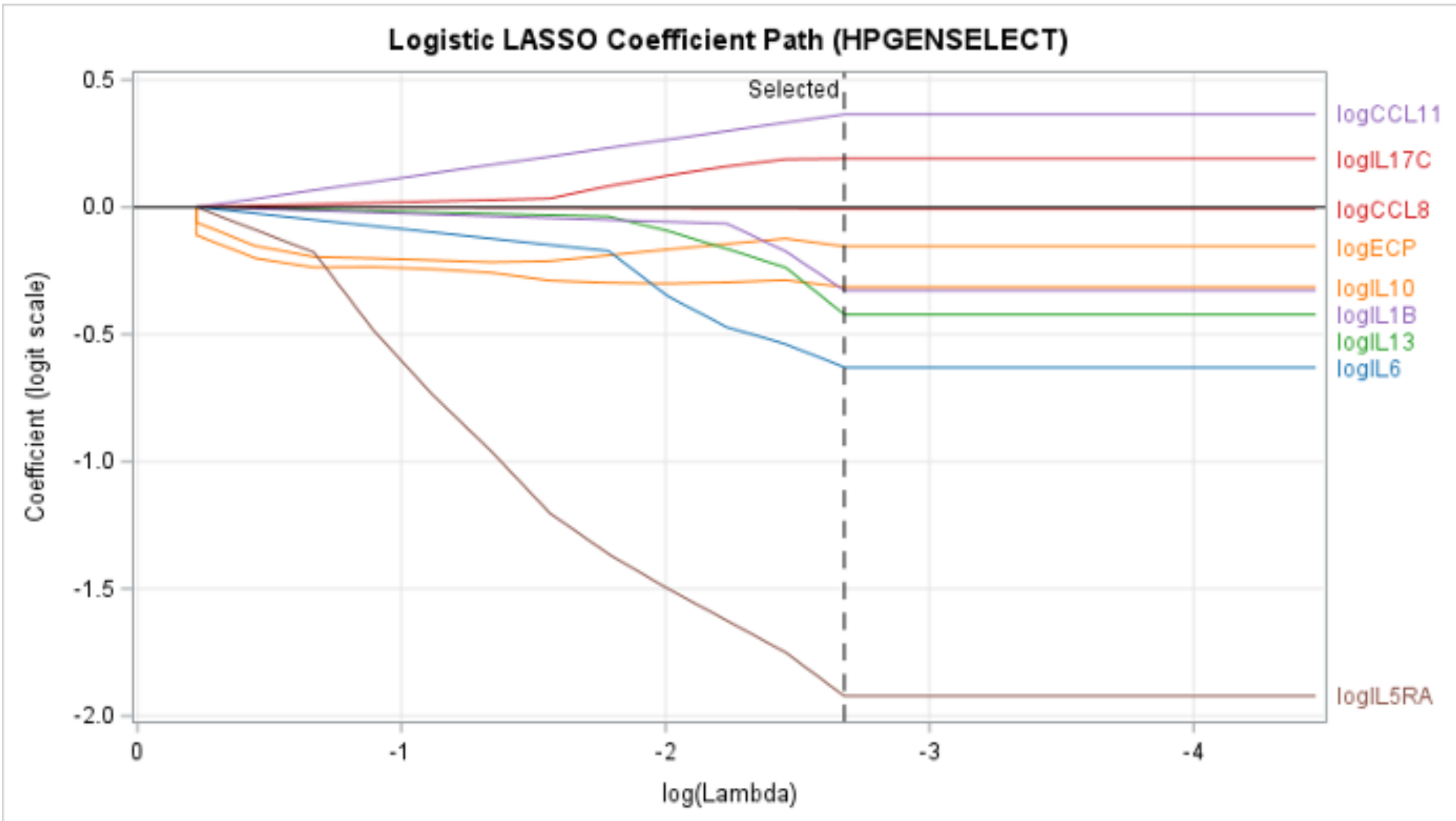

Logistic LASSO Coefficient Path (HPGENSELECT)
Selected
0.5
0.0
-0.5
-1.0
-1.5
-2.0
Coefficient (logit scale)
0
-1
-2
-3
-4
log(Lambda)
logCCL11
logIL17C
logCCL8
logECP
logIL10
logIL1B
logIL13
logIL6
logIL5RA

B.

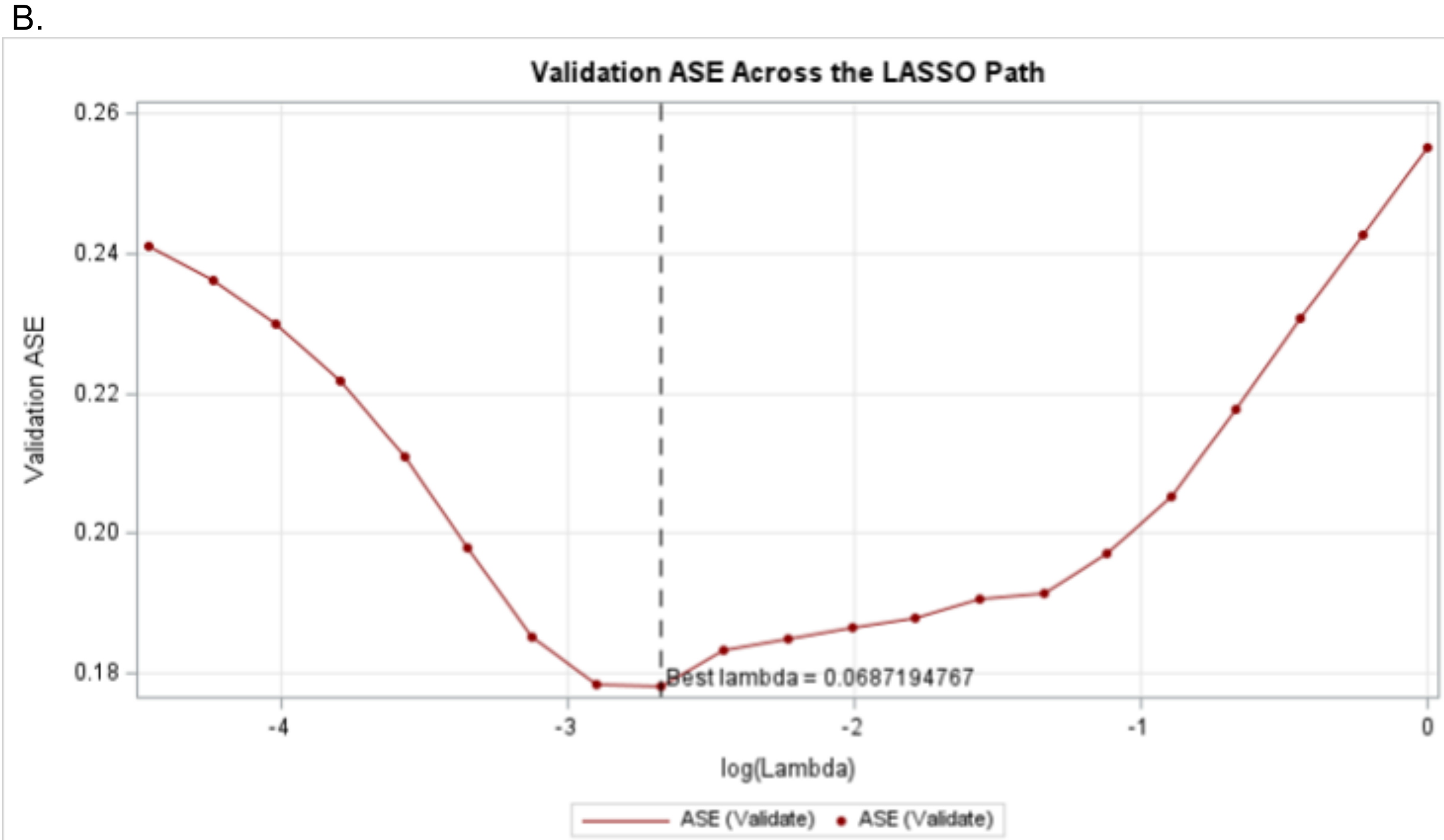

Validation ASE Across the LASSO Path
0.26
0.24
0.22
0.20
0.18
Validation ASE
Best lambda = 0.0687194767
-4
-3
-2
-1
0
log(Lambda)
ASE (Validate)
ASE (Validate)
