# Supplementary material for: Beyond type 2 inflammation: An innate–myeloid axis is linked to recurrence of chronic rhinosinusitis with nasal polyps
Source: J Allergy Clin Immunol. Author manuscript; Available in PMC 2026 Sep 24. (PMC13606457; doi:10.1016/j.jaci.2026.07.018)
Supplement: Supplementary file 2 [file NIHMS2203575-supplement-MMC4.pdf]

A.

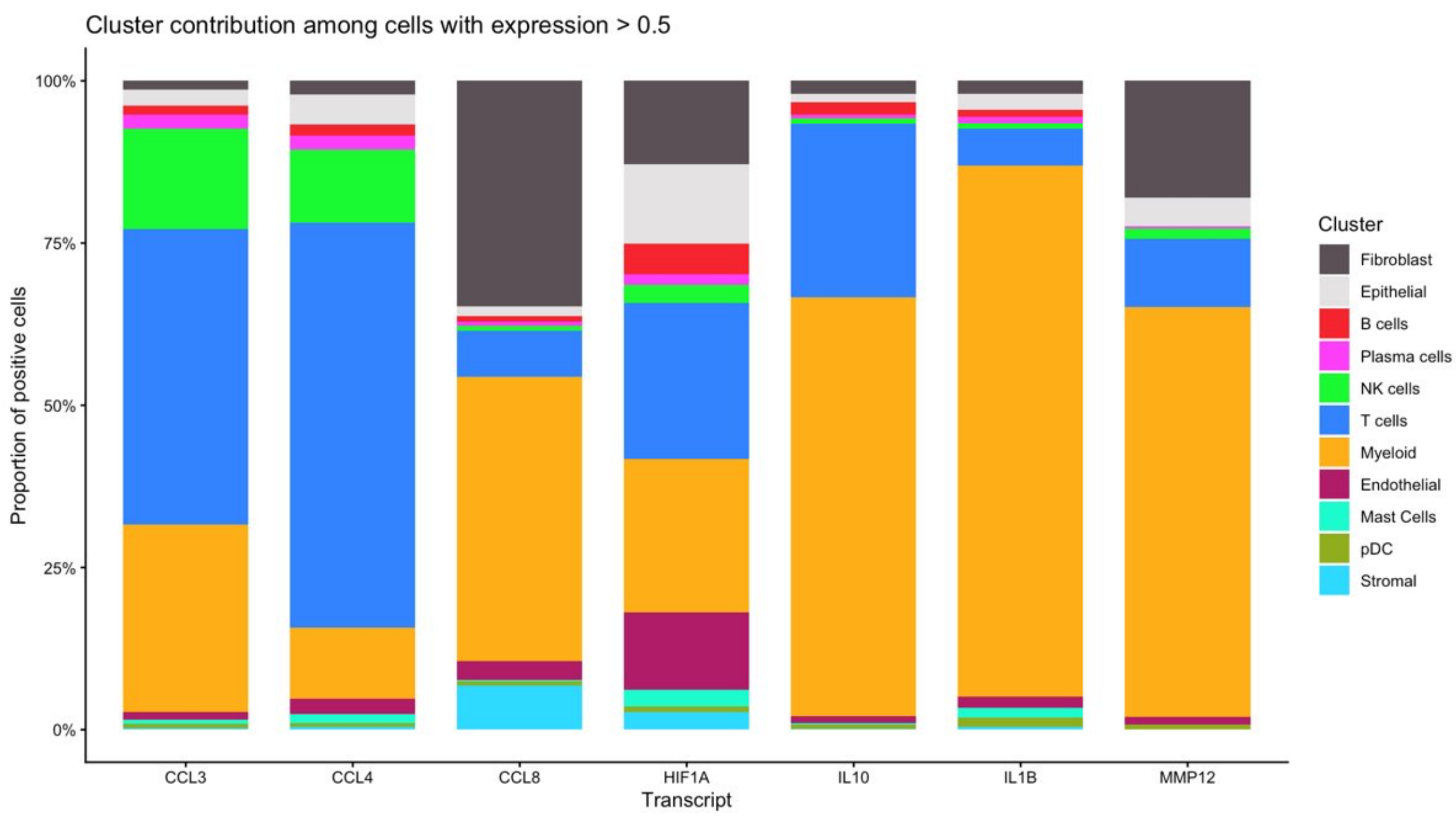
Cluster contribution among cells with expression > 0.5
Proportion of positive cells
100%
75%
50%
25%
0%
CCL3
CCL4
CCL8
HIF1A
IL10
IL1B
MMP12
Transcript
Cluster
Fibroblast
Epithelial
B cells
Plasma cells
NK cells
T cells
Myeloid
Endothelial
Mast Cells
pDC
Stromal

B.

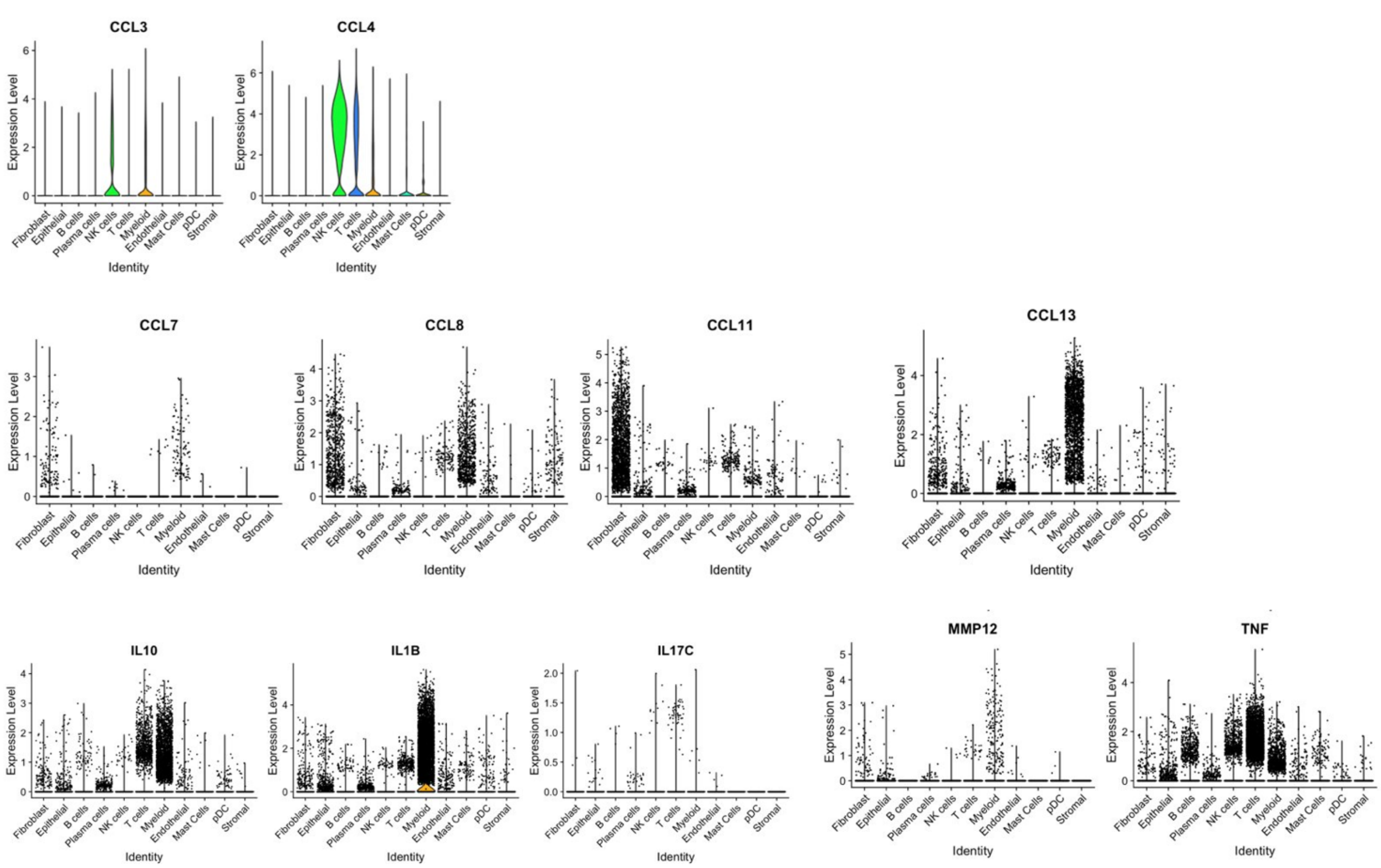
CCL3
CCL4
CCL7
CCL8
CCL11
CCL13
IL10
IL1B
IL17C
MMP12
TNF
Expression Level
Identity
Fibroblast
Epithelial
B cells
Plasma cells
NK cells
T cells
Myeloid
Endothelial
Mast Cells
pDC
Stromal
