# Supplementary material for: Beyond type 2 inflammation: An innate–myeloid axis is linked to recurrence of chronic rhinosinusitis with nasal polyps
Source: J Allergy Clin Immunol. Author manuscript; Available in PMC 2026 Sep 24. (PMC13606457; doi:10.1016/j.jaci.2026.07.018)
Supplement: Supplementary file 3 [file NIHMS2203575-supplement-MMC5.docx]

**Supplemental Table E1. Comprehensive mediator list.**
Complete list of all 69 cytokines, chemokines, and inflammatory mediators measured in nasal polyp tissue or nasal fluid across both cohorts via Olink and ELISA^1^.

**Supplemental Table E2. Patient outcomes pre-dupilumab treatment and two months on dupilumab treatment.**

**Supplemental Figure E1A: Logistic LASSO Coefficient Path using HPGENSELECT procedure.** Nine tissue biomarkers linked to rapid CRSwNP recurrence were identified: log CCL8, log CCL11, log IL-6, log IL-17C, log IL-1β, log IL-5Rα, log ECP, log IL-10, and log IL-13.

**Supplemental Figure E1B: Validation ASE across the LASSO path.** Cross-validation λ minimum was 0.0687.

**Supplemental Figure E2:** **Nasal polyp levels of other mediators that are predictive of CRSwNP recurrence.** Nasal polyp tissue concentrations of selected recurrence-associated mediators in patients with NSAID-ERD or aspirin-tolerant disease (NT-CRSwNP) who experienced rapid or slow/no post-endoscopic sinus surgery recurrence are shown. Selected chemokines (**A**) and mediators associated with signaling/receptors (**B**). Data are shown as medians and interquartile ranges. P values calculated by Wilcoxon signed-rank or t-test as appropriate, with *p<0.05; **p<0.01; ***p<0.001; ****p<0.0001.

**Supplemental Figure E3: Dupilumab-induced changes in other recurrence-associated mediators in nasal fluid.** Paired analyses of nasal fluid concentrations at baseline and after 2 months of dupilumab therapy (Month 2) in 17 CRSwNP patients. Shown are selected mediators identified as predictors of post-surgical recurrence including chemokines (**A**) and mediators associated with signaling/receptors (**B**). Horizontal lines indicate medians; boxes, interquartile range; whiskers, 10th–90th percentiles; P values from paired t-test or Wilcoxon signed-rank test, with *p<0.05; **p<0.01; ***p<0.001.

**Supplemental Figure E4: Single cell RNA-seq expression of mediators associated with CRSwNP recurrence.** Proportion of cells expressing CCL3, CCL4, CCL8, HIF1A, IL1B, MMP12, and IL10 transcript (**A**). Expression of CCL3, CCL4, CCL7, CCL8, CCL11, CCL13, IL10, IL1β, IL17C, MMP12, and TNF in each cell cluster in violin plots **(B)**.

**Supplemental Figure E5:** **IL-5Rα and IL-1β as predictors for CRSwNP recurrence.** Using LASSO regression followed by multivariable logistic regression to create a final model to predict CRSwNP recurrence, we identified logIL-5Rα and logIL-1β as most predictive for recurrence, with an AUC with 95%CI of 0.85 (95% CI of 0.77-0.93) (**A**). Logistic regression analysis was repeated, adjusting for the disease status (NSAID-ERD vs NT-CRSwNP) the same (AUC with 95%CI was 0.85 (0.77-0.93) (**B**).

**Supplemental Figure E6: Correlation of nasal lysate IL-5Rα and eosinophilic cationic protein (ECP), CD79B, and epidermal growth factor (EGF).** Spearman’s rank correlation.

**Supplemental Figure E7: Type 2 cytokines that are not increased in participants with CRSwNP recurrence.** Nasal polyp concentrations of IL-5, TSLP, and IL-33 in participants with NSAID-ERD or aspirin-tolerant disease (NT-CRSwNP) who experienced rapid or slow/no post-operative recurrence.
