# Supplementary material for: Beyond type 2 inflammation: An innate–myeloid axis is linked to recurrence of chronic rhinosinusitis with nasal polyps
Source: J Allergy Clin Immunol. Author manuscript; Available in PMC 2026 Sep 24. (PMC13606457; doi:10.1016/j.jaci.2026.07.018)
Supplement: Supplementary file 4 [file NIHMS2203575-supplement-Supplemental_Table_E1.docx]

**Supplemental Table E1. Comprehensive mediator list.**
Complete list of all 69 cytokines, chemokines, and inflammatory mediators measured in nasal polyp tissue or nasal fluid across both cohorts via Olink and ELISA^1^.

**Proteins**

| AREG | IL-5 |
| --- | --- |
| CCL2 | IL-6 |
| CCL3 | IL-7 |
| CCL4 | IL-10 |
| CCL7 | IL-13 |
| CCL8 | IL-15 |
| CCL11 | IL-18 |
| CCL13 | IL-25 |
| CCL17 | IL-27 |
| CCL19 | IL-33 |
| CCL20 | IL-12A and B |
| CASP8 | IL-17A |
| CD36 | IL-17C |
| CD79B | IL-17F |
| CSF1 | IL-1β |
| CSF2 | IL-5Rα |
| CSF3 | KITLG |
| CSF2RB | LIF |
| CXCL1 | LTA |
| CXCL8 | MMP-1 |
| CXCL9 | MMP-12 |
| CXCL10 | Neutrophil Elastase^1^ |
| CXCL11 | OLR1 |
| CXCL12 | OSM |
| CXCL13 | TGFα |
| ECP^1^ | TGFβ1 |
| EGF | TNF-α |
| FLT3LG | TNFRSF4 |
| HGF | TNFSF10 |
| HIF-1α | TNFSF12 |
| IFN-α2 | TNFSF14 |
| IFNγ | TSLP |
| IgE^1^ | VEGFA |
| IL-2 | VEGFD |
| IL-4 |  |
