# Supplementary material for: Beyond type 2 inflammation: An innate–myeloid axis is linked to recurrence of chronic rhinosinusitis with nasal polyps
Source: J Allergy Clin Immunol. Author manuscript; Available in PMC 2026 Sep 24. (PMC13606457; doi:10.1016/j.jaci.2026.07.018)
Supplement: Supplementary file 5 [file NIHMS2203575-supplement-Supplemental_Table_E2.docx]

**Supplemental Table E2: Patient outcomes pre-dupilumab and two months on dupilumab treatment.**

|  | Pre-Dupilumab (N = 17) | On-Dupilumab (N = 17) | p-value |
| --- | --- | --- | --- |
| SNOT-22 Score | 40.0 (26.5-60.0) | 18.0 (8.0-27.5) | **<0.0001** |
| UPSIT | 10.0 (10.0-20.5) | 28.0 (14.5-35.0) | **<0.001** |
| Peak Nasal Inspiratory Flow (PNIF) | 90.0 (52.5-115.0) | 135.0 (90.0-160.0) | **<0.01** |
| Total Nasal Polyp Score | 6.0 (3.3-6.0) | 2.0 (1.0-5.8) | **=0.02** |

Numerical variables were reported with Median (Q1-Q3) unless otherwise noted.
